# Recent developments in classical density modification

**DOI:** 10.1107/S090744490903947X

**Published:** 2010-03-24

**Authors:** Kevin Cowtan

**Affiliations:** a Department of Chemistry, University of York, Heslington, York YO10 5DD, England

**Keywords:** density modification, phase improvement, *Parrot*

## Abstract

Classical density-modification techniques (as opposed to statistical approaches) offer a computationally cheap method for improving phase estimates in order to provide a good electron-density map for model building. The rise of statistical methods has lead to a shift in focus away from the classical approaches; as a result, some recent developments have not made their way into classical density-modification software. This paper describes the application of some recent tech­niques, including most importantly the use of prior phase information in the likelihood estimation of phase errors within a classical density-modification framework. The resulting software gives significantly better results than comparable classical methods, while remaining nearly two orders of magnitude faster than statistical methods.

## Abstract 2

Several new methods are evaluated for use in the improvement of experimental phases in the framework of a classical density-modification calculation. These methods have been implemented in a new computer program, *Parrot*.

## 1. Background

Phase improvement by density modification has become a routine part of the process of structure solution using experimental phases and is often also used after molecular replacement. There are two families of approaches to density modification: ‘classical’ methods, which iterate modifications to the electron-density map in real space with the reintroduction of the experimental observations in reciprocal space, and ‘statistical’ methods, which construct a probability distribution for the electron-density values as a function of position in real space and transform this distribution to obtain a probability distribution for the phases in reciprocal space.

### 1.1. Classical density modification

Classical density-modification methods have provided a convenient tool for the rapid calculation of ‘improved’ electron-density maps for more than 15 years and have been employed in a number of forms, with the common feature of alternating steps being performed in real and reciprocal space. The calculation commonly follows the following pattern.

Starting with a set of experimentally observed structure-factor magnitudes and estimated phase probability distributions, a ‘best’ electron-density map is calculated using the centroid of the phase probability distribution to provide a phase and weight for the structure-factor magnitude.

This initial electron-density map is then modified to make it conform more closely to the features expected of a well phased electron-density map. The most common modifications are as follows.

(i) Solvent flattening (Wang, 1985 ▶). Features in the solvent region are flattened under the assumption that noise arising from errors in the phases provides a significant contribution to such features.
(ii) Histogram matching (Zhang *et al.*, 1997 ▶). The histogram of electron-density values for a well phased map differs from the histogram for a randomly phased map. The application of a nonlinear rescaling to the electron density allows the electron-density map to be modified so that its histogram looks more like that of a well phased map. This process tends to sharpen electron-density peaks and suppress negative density.
(iii) Noncrystallographic symmetry (NCS) averaging. In cases where there are several copies of a molecule in the asymmetric unit, the related electron-density values between the molecules may be averaged to improve the signal-to-noise ratio and impose restraints on the phase values.

The modified map is then back-transformed, leading to a new set of Fourier coefficients which differ in both magnitude and phase from those used to calculate the initial map. An error estimate is calculated for each phase, usually on the basis of how well the modified magnitudes match the observed values in a particular resolution shell. This error estimate is used to construct a phase probability distribution centred about the modified phase.

This phase probability is multiplied by the phase probability distribution from the experimental phasing to provide an updated distribution. The new distribution can be used to calculate an electron-density map for model building or can be used to start a new cycle of density modification.

This basic scheme has been implemented with some refinements in the *DM* (Cowtan *et al.*, 2001 ▶) and *SOLOMON* (Abrahams & Leslie, 1996 ▶) software, with some variations, as well as in many other packages. The *DM* software initially implemented solvent flattening, histogram matching and NCS averaging, along with likelihood error estimation using the σ_A_ method (Read, 1986 ▶). The *SOLOMON* software pioneered the use of weighted NCS averaging and also the use of solvent flipping to reduce bias, which was later implemented in *DM* in the form of the ‘perturbation’ gamma correction (Abrahams, 1997 ▶; Cowtan, 1999 ▶).

One distinct technique which is not described here is the use of density modification for resolution extrapolation beyond the limit of the observed data. Pioneered by Caliandro *et al.* (2005 ▶) and more widely used in the software of Sheldrick (Usón *et al.*, 2007 ▶), this approach can provide significant additional phase improvement, especially when the data already extend to better than 2 Å resolution.

### 1.2. Statistical density modification

Statistical density-modification methods provide a more sound theoretical basis to the problem of phase improvement and as a result reduce the problems of bias associated with classical density-modification methods. This improvement is achieved in two ways.

(i) By the expression of the additional information to be introduced to the electron-density map in terms of probability distributions and then carrying those distributions into reciprocal space, rather than working with a single map representing a single sample from the phase probability distributions.
(ii) By weakening the link between the additional information to be introduced and the initial phases, thus reducing the bias introduced in a single cycle of phase improvement. Since the current centroid map is not used as the basis for phase improvement, the phase probability distributions from which the centroid map is derived are not directly included in the new phase information incorporated during a single density-modification cycle. The only way in which the current phases are used is in the classification of the asymmetric unit into regions of different density types, *e.g.* solvent and protein.

The result of these two changes is that statistical density-modification techniques lead to reduced phase bias and more realistic estimates of the figures of merit.

The resulting method has been implemented in the *RESOLVE* software (Terwilliger, 1999 ▶). In addition to its application to conventional density-modification problems, it has been particularly effective in removing bias from maps phased from an atomic model through the ‘prime-and-switch’ approach (Terwilliger, 2004 ▶). An alternative implementation in a program called *Pirate* (Cowtan, 2000 ▶) has been employed successfully in a number of cases, but delivers poor results in other cases for reasons which have yet to be determined.

### 1.3. Limitations of current methods

Statistical phase-improvement methods, and in particular the *RESOLVE* software, have made a substantial contribution to the field of phase improvement, significantly reducing the problem of bias and additionally providing tools for removing bias from existing phasing. Current implementations are also highly automated, making them particularly suitable for use in structure-solution pipelines. The only significant limitation of these approaches is the computational overhead, with calculations taking minutes rather than seconds.

During the rise of statistical methods, classical density-modification techniques have been neglected to some extent, most notably in the implementation of automation features. However, another effect of this neglect has been a failure to implement a number of algorithms which are now routine in other steps of the structure-solution pipeline.

The aim of this work is to produce an up-to-date classical density-modification method that is updated to incorporate both automation features and the latest applicable algorithms. Where it has been convenient to do so, direct comparisons have been made to demonstrate the effect of updating each step of the process. The resulting algorithm retains the speed benefits of classical density-modification techniques; it is hoped that this will render it suitable for interactive use from within graphical model-building programs, for example in *Coot* (Emsley & Cowtan, 2004 ▶).

## 2. Methods

The density-modification algorithm described here follows closely the outline of classical methods and in particularly the approach implemented in the *DM* software; however, the detailed implementation of some of the steps has been altered. Specifically, the calculation consists of some data-preparation steps followed by a loop in which the data manipulations occur successively in real and reciprocal space. The calculation involves the following steps.

(i) Perform an anisotropy correction on the input structure factors.
(ii) (Optional) Estimate the solvent content from the sequence.
(iii) (Optional) Calculate NCS operators from heavy-atom coordinates or from an atomic model.
(iv) Cycle over the following steps a specified number of times.
  (1) Simulate electron-density histograms for the ordered region of the asymmetric unit using a known structure.
  (2) Calculate an electron-density map using centroid phases and weights based on the current phase probability distributions.
  (3) Calculate a solvent mask covering the required volume of the unit cell.
  (4) (Optional) Prepare an NCS map consisting of the contributions from other NCS copies to each position in the asymmetric unit.
  (5) Prepare a perturbed map from the initial map by adding a small random signal.
  (6) Density-modify the initial map by applying the NCS contributions, solvent flattening and histogram matching.
  (7) Density-modify the perturbed map by applying the NCS contributions, solvent flattening and histogram matching.
  (8) Compare the two modified maps to estimate the gamma correction required.
  (9) Apply the gamma correction to the modified unperturbed map.
  (10) Back-transform to obtain a set of modified magnitudes and phases.
  (11) Calculate an error model by optimizing the likelihood of the observed data given the calculated data and error model parameters (*i.e.* a σ_A_-type calculation).
  (12) Use the error model to calculate updated Hendrickson–Lattman coefficients and 2*mF*_o_ − *DF*_c_-type map coefficients.

The general steps of the calculation are very similar to those employed in the *DM* software. In particular, the gamma-correction calculation is the perturbation gamma method from Cowtan (1999 ▶), with the exception that the perturbation calculation is performed in real rather than reciprocal space. The solvent-flattening and histogram-matching calculations are identical to those described by Zhang *et al.* (1997 ▶). The solvent mask-determination algorithm is identical to that employed by Abrahams & Leslie (1996 ▶) in the *SOLOMON* software.

The principal differences to the methods mentioned above are as follows.

(i) Problem-specific histogram simulation using a known structure.
(ii) Use of prior phase information in the calculation of figures of merit and map coefficients.
(iii) Application of anisotropy correction to the data.
(iv) Pairwise weighted noncrystallographic symmetry averaging.

These will be discussed in turn in the following sections.

### 2.1. Problem-specific histogram simulation from a known structure

The implementation of histogram matching in the *DM* software depended on the use of a standard library of protein histograms calculated from known structures. However, the electron-density histogram is strongly dependent on both the resolution and the Wilson *B* factor of the data. As a result, in order for this procedure to work it was necessary to rescale the data to match the *B* factor of the histogram data set before calculating the electron-density map. For simplicity, the overall Wilson *B* factor was removed from the source data before calculating the reference histogram libraries (*i.e.* using maps for a pseudo-stationary atom structure) and the working data were also sharpened using a method documented by Cowtan & Main (1998 ▶).

The use of a sharpened map potentially introduces additional noise arising from the lower signal-to-noise ratio and poorer phasing of the high-resolution reflections. A better approach is to calculate histograms appropriate to the current problem by matching the resolution and temperature factor of the source data sets from which the histogram is obtained to those of the data from the structure to be solved. The modified source data will then yield histograms that are appropriate to the current problem. (If desired, the data for the unknown structure can also be sharpened or smoothed beforehand.)

This approach has been implemented by providing a solved reference structure with observed structure factors and calculated phases from which the software can generate an appropriate histogram library on the fly. The choice of the reference structure does not appear to be critical for normal problems; however, the user can optionally provide their own reference structure if there is a good reason to do so.

The figures of merit may also vary systematically as a function of resolution: they will normally be lower at high resolutions. If this contribution is ignored, the electron-density histogram for the reference structure will be systematically sharper than the electron-density histogram for the work structure. Using an over-sharp histogram for histogram matching will tend to up-weight the high-resolution terms, for which the phases are usually worst.

The protein density-histogram library is therefore calculated in the following way. The structure factors and phases from the refined model for the reference structure are read into the program, along with the structure factors for the unsolved work structure. The resolution of the reference structure is truncated to match the work structure. The reference-structure data are rescaled with a resolution-dependent scale function (using a smooth-spline scaling following the method of Cowtan, 2002 ▶) to match the scale of the work structure data; this resolution-dependent scaling effectively matches the Wilson *B* factors. The effect of the resolution-dependence of the figures of merit is also simulated by creating synthetic figures of merit for the rescaled reference structure factors, matching the resolution distribution for the work structure factors. These synthetic figures of merit are used as weights in the calculation of the electron-density map for the reference structure.

The known atomic model for the reference structure is then used to calculate a solvent mask and electron-density histograms from the protein region of the simulated map. The resulting histogram may then be used as a target histogram for histogram matching the work map, following the method of Zhang *et al.* (1997 ▶).

### 2.2. Use of prior phase information in the calculation of figures of merit and map coefficients

After the application of techniques such as solvent flattening and histogram matching to the electron density, an inverse Fourier transform is used to obtain a new set of magnitudes and phases. These are then used to update the phase probability distributions arising from the original experimental phasing calculation.

Most previous density-modification algorithms, including *DM*, *SOLOMON* and *CNS* (Cowtan *et al.*, 2001 ▶; Abrahams & Leslie, 1996 ▶; Brünger *et al.*, 1998 ▶), have adopted a two-stage approach to this problem. In the first step, an estimate of the reliability of the modified phases is made on the basis of the agreement between the modified magnitudes and the ob­served structure factors. The reasoning behind this approach comes from analogy with the problem of calculating map coefficients using a partial structure including both errors and missing atoms and is based on the fact that the size of the discrepancy in the structure-factor magnitudes is a good indicator of the error in the phases.

Once an estimate of the error in the phases has been obtained, a phase probability distribution is constructed from the modified phase and estimated error. This phase probability distribution is multiplied by the experimental phase probability distribution to provide an updated distribution. [The distributions are usually represented in terms of Hendrickson–Lattman coefficients (Hendrickson & Lattman, 1970 ▶) and so this multiplication is performed as a simple addition of co­efficients.] Map coefficients may also be calculated for ‘best’ and ‘difference’ electron-density maps.

To be more specific, the true structure factor is accounted for by two components: a portion of the calculated structure factor (reduced in magnitude because of the errors in the model) and an unknown portion which is represented by a two-dimensional Gaussian in the Argand diagram centred on the reduced calculated structure factor. This approach was developed by Read (1986 ▶) (using the terms *D* and σ_A_ for the scale term and the width of the Gaussian). The error and scale terms are related and are calculated in resolution shells. An alternative implementation using spline coefficients to provide a smooth variation with resolution has been described in Cowtan (2002 ▶) (using the terms *s* and ω for the scale term and the width of the Gaussian).

The approach adopted here is to include the prior experimental phase probability distribution into the calculation of the phase probability distribution for the modified phase and in doing so obtain improved estimates of the scale and error terms. In addition, the updated phase probability distribution and the electron-density map coefficients are obtained directly as part of the same calculation.

**Figure 1:**
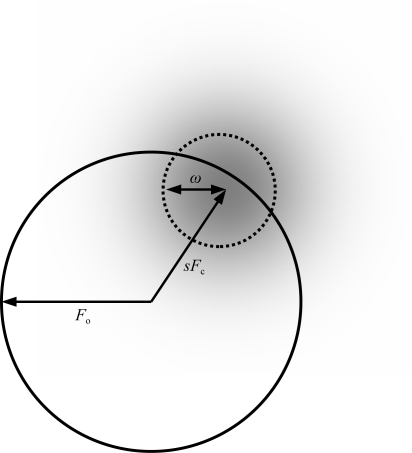
The terms *F*_o_, *F*_c_, *s* and ω describe the observed and calculated structure factor, the scale factor and the radius of the Gaussian error term in the Argand diagram. The shading represents the Gaussian probability distribution centred on *sF*_c_.

The method followed is almost identical to that of Cowtan (2005 ▶), with the following difference. The underlying equation for the probability of a phase is given by an equation which includes both the contribution from the calculated structure factor (scaled by a factor *s* with a Gaussian error term of width ω; see Fig. 1 ▶) and the contribution from the Hendrickson–Lattman coefficients, 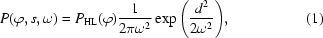
where *d* is the difference between the vectors (*sF*_c_, ϕ_c_) and (*F*_obs_, ϕ), *i.e. d* ^2^ = |*F*_o_|^2^ + *s* ^2^|*F*_c_|^2^ − 2|*F*_o_|*s*|*F*_c_|cos(ϕ − ϕ_c_).

This neglects the contribution of the error in the observed *F*, *i.e.* σ*_F_*. In the previous approach, σ*_F_* was used to increment the width of the Gaussian error term ω. This is no longer strictly correct, although when the phase errors in the model dominate (for example in the case of density modification, as contrasted with the very final stages of refinement) it is a good approximation.

In order to estimate *s* and ω, the unknown phase must be integrated out. Integrating the above expression and eliminating constant factors gives rise to 
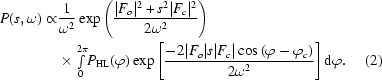
The logarithm of this function and its derivatives, summed over all reflections by resolution, are evaluated and used to determine maximum-likelihood estimates for *s* and ω.

As with the likelihood refinement target adopted by Pannu *et al.* (1998 ▶), the difference map (*i.e. mF*_o_ − *DF*_c_-like) co­efficients may be obtained by calculating the gradient of the logarithm of the likelihood function (2) with respect to the calculated structure factor and adjusting the scale to match that of the centroid map. The ‘best’ (*i.e.* 2*mF*_o_ − *DF*_c_-like) map is obtained by adding the centroid and difference maps. This map is used as a starting point for subsequent cycles of density modification.

### 2.3. Application of an anisotropy correction to the data

Anisotropy in the X-ray diffraction data can lead to similar groups of atoms which look very different in the electron-density map depending on their orientation with respect to the anisotropy of the data. This can affect the density-modification calculation in a number of ways, most notably in estimation of the solvent envelope and in the electron-density histogram of the data. The effects of anisotropy can be reduced by applying an anisotropy correction to the data to enhance the structure factors along directions in which they are weaker (although this does not correct for an anisotropic resolution limit) and this technique has been applied effectively even without an atomic model in programs such as *Phaser* (McCoy, 2007 ▶; Read, 2008 ▶).

An anisotropy correction has been implemented to adjust the input structure factors before the calculation of the first electron-density map. To estimate the anisotropy of the input data, *E* values are calculated from the observed structure factors. An anisotropic Gaussian is then determined which best fits the *E* values to the expected value of 1. In order to maintain the speed of the calculation, the scale is estimated by fitting a general quadratic in three dimensions to the logarithm of the *E* values, which is a linear rather than non­linear calculation and thus does not require iteration. The anisotropy correction is therefore obtained by minimizing the residual 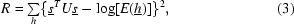
where 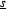 is the reciprocal orthogonal coordinate corresponding to the reflection index 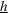 and *U* is the symmetric matrix of anisotropy coefficients.

This approach does not account for the experimental uncertainties and gives different weights to reflections of different magnitudes, but tests using both simulated and real data give similar results to the more thorough approach adopted in *REFMAC* (Murshudov *et al.*, 1997 ▶).

### 2.4. Pairwise weighted noncrystallographic symmetry averaging

The concept of weighted NCS averaging was introduced by Abrahams & Leslie (1996 ▶) to deal with a case in which different parts of the structure obeyed the NCS relationships to different degrees. This was achieved by use of a ‘weighted averaging mask’; instead of having values of 0 (for unrelated regions of the map) or 1 (for NCS-related regions of the map), Abrahams’ mask could take values in a continuous range between 0 and 1 representing different levels of agreement. In regions where the mask value was less than 1, the weight of the NCS-related density would be less than the weight of the original density at that position in the map.

The approach described here extends this work by the introduction of multiple masks, with one mask for each pair of NCS-related density regions. Thus, in the case of threefold symmetry between molecules *A*, *B* and *C* there are six masks: those relating molecules *A*–*B*, *A*–*C*, *B*–*A*, *B*–*C*, *C*–*A* and *C*–*B*. This allows for the case where some pairs of molecules may be more similar than others. For example, if each of the molecules *A*, *B* and *C* have two domains, α and β, both domains may be similar in molecules *A* and *B* but domain β may be missing in molecule *C*. In this case a different mask is required when averaging between molecules *A* and *B* as opposed to averaging either of these with molecule *C*.

Previous implementations (*e.g.* Vellieux *et al.*, 1995 ▶) have calculated a mask covering the NCS-related region at the beginning of the density-modification calculation and then stored this mask for use during the rest of the calculation; however, with so many masks this becomes inconvenient. Instead, the masks are calculated on the fly as they are required, using a highly optimized FFT-based approach.

To calculate the mask relating molecules *A* and *B*, two maps are calculated covering a spherical region of at least four asymmetric unit volumes about the estimated centre of molecule *A*. The first map contains the unrotated density for molecule *A* and the second contains the density from molecule *B* rotated back into the same orientation as molecule *A*. Both these maps are subsampled to 1/3 of the sampling (*i.e.* three times the grid spacing) of the initial electron-density map in order to reduce the computational overhead.

The local correlation between the two maps will be used to determine which regions obey the NCS and is calculated by an FFT to further reduce the computational overhead. By default, the local correlation is calculated over a sphere of 6 Å radius about each point in the map. Given the two subsampled maps ρ_*A*_ and ρ_*B*_, the correlation function *C*_local_ is given by the formula
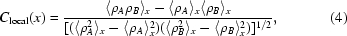
where 
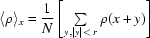
and *N* is the number of grid points within a sphere of radius *r*. Each of the local averages can be calculated by the convolution theorem, requiring two FFTs (plus one additional FFT to calculate the Fourier transform of the spherical mask), giving a total of 11 FFTs. Note that these FFTs are not calculated over the unit cell, as would normally be the case, but rather over a box containing the subsampled grid covering the region of interest. Since these maps are nonrepeating, the map must be padded with smoothed values at the edges to avoid introducing spurious high-resolution terms during the FFTs.

The resulting map gives values for the local correlation of the NCS-related regions for every point in the region of interest. The next step is to obtain some estimate of the significance of the correlation values. To do this, a similar local correlation map, calculated between two unrelated regions of density, is used to determine the expected standard deviation σ_C_ of the local correlation values from zero (*i.e.* the mean correlation for unrelated density regions).

This standard deviation is then used to convert the local correlation map into a weighted mask function *w*_ncs_(x), according to the formula
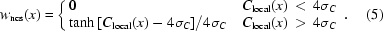
This gives mask values increasing from 0 towards 1 as the local correlation increases above 4σ_C_.

This weighted mask is still sampled on the coarse grid. The final step is to interpolate the mask values by trilinear interpolation from the coarse grid back onto the original map grid, giving a mask covering the electron density of molecule *A* on the same grid as molecule *A*.

## 3. Results

The approaches described in this paper have been implemented in *Parrot*, an automated density-modification program. Where it has been simple to do so both the existing and new approaches have been implemented, allowing a direct comparison of the benefits of the new technique that is independent of any other implementation differences. For the remaining cases, some limited inferences may be drawn by comparison of the results from *Parrot* against the results from the earlier *DM* software. The new techniques described in the previous section will be considered in turn.

The techniques are compared here in terms of the correlation between the density-modified electron-density map and the electron density calculated from the refined structure, with a value of 1 indicating perfect phases and 0 indicating random phases. This approach has an advantage over using a simple or weighted mean phase error in that it is insensitive to changes in the phases of very weak reflections which do not affect the map significantly. (The weighted mean phase error and *E*-map correlation were also investigated and show similar behaviour to the map correlations presented here in most cases.)

### 3.1. Problem-specific histogram simulation from a known structure

The use of a problem-specific histogram library is the only technique which is implemented in *Parrot*; thus, to compare the results with the use of a standard library for a stationary-atom structure the results of *Parrot* (with all the other new features excluded) must be compared against the results of the *DM* software. The results may therefore be confounded by other differences in the software. The most notable of these is the different solvent mask-determination algorithm.

The map correlations for the basic *Parrot* calculation were compared with the map correlations for the *DM* calculation using 58 experimentally phased structures from the JCSG data archive (Joint Center for Structural Genomics, 2006 ▶) spanning the resolution range 1.4–3.2 Å. The phasing from the original JCSG structure solution using either MAD or SAD data was used as a starting point for the density-modification tests. In some cases multiple phasing calculations had been run; in this case the phasing run which produced the electron-density map with the greatest contrast (given by the r.m.s.d. of the local r.m.s.d., which is a crude indicator of map quality) was used. A list of the JCSG data sets and the corresponding phasing files used has been deposited as supplementary material to this paper^1^.

**Figure 2:**
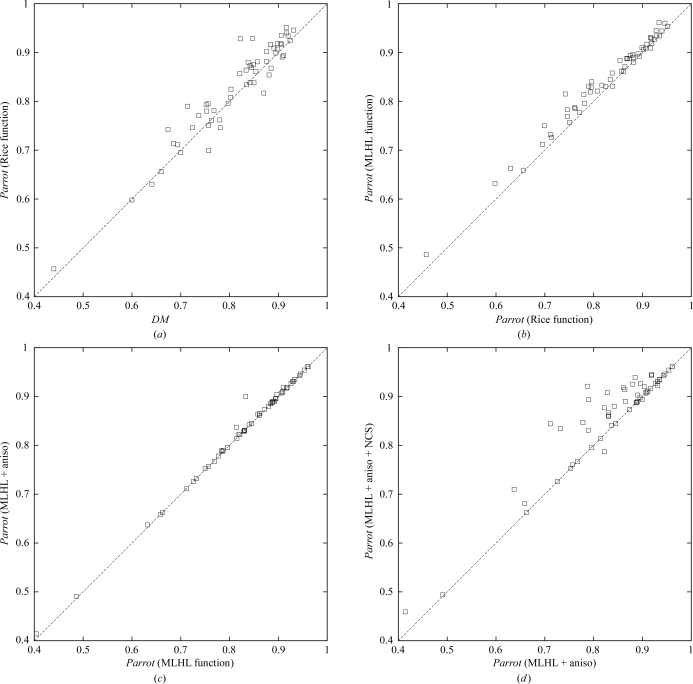
Mean map correlation calculated of a range of JCSG data sets with using different density-modification programs and options. (*a*) *Parrot* with no new features compared with *DM*. (*b*) *Parrot* with MLHL likelihood function compared with the Rice function. (*c*) As (*b*), with anisotropy correction compared with no anisotropy correction. (*d*) As (*c*), with automated NCS averaging compared with no NCS averaging.

For each structure, the *Parrot* result is plotted against the *DM* result as a scatter plot; thus, any point falling above the diagonal line *y* = *x* represents a case where *Parrot* gives a better map than *DM*. The resulting plot is shown in Fig. 2 ▶(*a*).

Note that the new implementation in *Parrot*, performing a similar calculation to *DM* with the exception of the mask-calculation algorithm and the problem-specific histogram libraries, gives broadly similar results. Each program performs better on some structures, but the mean map correlation over all the structures is higher for *Parrot* (0.771 for *Parrot versus* 0.759 for *DM*). There is, however, no obvious indication (*e.g.* dependence on resolution or solvent content) why one program works better than the other in any individual case.

### 3.2. Use of prior phase information in the calculation of figures of merit and map coefficients

In order to test the use of prior phase information, the results of *Parrot* were compared using both the new likelihood function incorporating the prior phase information and the Rice-function implementation (*i.e.* the same method used in *DM*). The latter set of results are the *Parrot* results from the previous section. The results for the new likelihood function are plotted against the results for the old function and the resulting plot is shown in Fig. 2 ▶(*b*).

Note that the results are improved in the majority of cases and in no case does the prior phasing leads to a significantly worse result. The mean map correlation over all the structures increases from 0.771 to 0.785.

One effect of the use of prior phase information in the estimation of errors in the modified structure factors may be the reduction of bias in the density-modification calculation. Without prior phase information, the modified phases may be over-weighted by the modified magnitudes matching the observed values, a state which can be achieved without necessarily fitting the phases correctly. With prior phase information, if the modified phases are wrong and some prior phase information is present in a resolution shell against which to compare them, then those phases will contribute to a higher error estimate. As a result, the problem of bias is reduced.

### 3.3. Application of an anisotropy correction to the data

The effect of the anisotropy correction was tested in the same way, comparing the previous set of results against the results with the same calculation performed using the anisotropy correction. The results for the anisotropy-corrected calculation are compared with the results from the uncorrected case and the resulting plot is shown in Fig. 2 ▶(*c*).

Note that in the majority of cases the correction makes no difference, but in a minority of cases there is a slight improvement in the results and in two cases the improvement is significant. The improvement occurs in cases where the anisotropy is large, although not all anisotropic data sets improve significantly. The results are never worse and the computational overhead is minimal.

### 3.4. Pairwise weighted noncrystallographic symmetry averaging

NCS averaging with a single (binary) averaging mask covering all related NCS copies of a molecule has not been implemented in *Parrot* and thus a direct comparison is not possible. Comparison to *DM* is confounded by the differences already noted in §[Sec sec3.1]3.1 and by the fact that averaging is not automated in *DM* and involves manual entry of the averaging operators. As a result, no empirical conclusions can be drawn concerning the benefits of pairwise weighted averaging in comparison to existing methods.

However, a comparison between the *Parrot* results with and without averaging is presented as a demonstration that the method works as an automated tool for improving electron-density maps. The map correlations from the automated NCS-averaging calculation are plotted against the results without averaging (from the previous test) and the resulting plot is shown in Fig. 2 ▶(*d*).

Note that in about half the cases shown the results are significantly improved: these are the cases where the NCS has been correctly determined from the heavy-atom coordinates. For the remaining cases no NCS is present or the NCS could not be identified. In four cases, incorrect NCS operators are determined; however, the weighted averaging mask procedure tends to down-weight the impact of incorrect NCS, so that in only one of these cases is the difference in map correlation significant.

### 3.5. Other comparisons

The amount of computation required for classical and statistical density-modification methods differs substantially. The *DM* calculation was very fast (a mean of 6 s per structure) and the *Parrot* calculation only slightly slower (a mean of 10 s per structure), while the statistical method of *Pirate* was approximately two orders of magnitude slower (a mean of 887 s per structure).

An important test of a density-modification technique is whether it allows an atomic model to be built into the resulting electron density. To this end, automated model-building calculations were performed using the *Buccaneer* model-building software (Cowtan, 2006 ▶) starting from the modified phases from each density-modification program in turn. After averaging over all the test cases to minimize variations arising from instabilities in the model-building calculation, the results were consistent with the mean map correlations reported earlier.

### 3.6. Future work

There is scope for further development of the methods devised here. There are no technical obstacles to implementation of resolution extrapolation beyond the limit of the observed data (Caliandro *et al.*, 2005 ▶; Usón *et al.*, 2007 ▶). The combination of resolution extrapolation with the likelihood-weighting methods described in §[Sec sec2.2]2.2 may or may not provide additional benefits.

Multi-crystal averaging, as currently implemented in the *DMMULTI* software, could also be implemented in *Parrot*. The greatest challenge here is one of automation; in particular the determination of cross-crystal averaging operators.

The speed of the program provides scope for various iterative and multi-start approaches, for example optimization of solvent content (as suggested by a referee) or a data-sharpening factor could be achieved with a suitably reliable indicator of the quality of the resulting map.

## 4. Conclusions

Classical density-modification techniques still have significant value. When updated to use the latest methods, in particular the use of prior phase information in the estimation of errors, they can be competitive or nearly competitive with statistical methods while requiring a fraction of the computation time. In addition, the implementation described here in the *Parrot* software appears to be robust when applied to data from different sources.

The speed of the approach described here lends itself to particular problems, including the fast assessment of experimental data at the beamline (in combination with automated phasing and fast model-building algorithms) or use in parallel hierarchical automation models in which many structure-solution pathways are explored in parallel.

## Supplementary Material

Supplementary material file. DOI: 10.1107/S090744490903947X/ba5136sup1.txt

## Footnotes

1 Supplementary material has been deposited in the IUCr electronic archive (Reference: BA5136). Services for accessing this material are described at the back of the journal.
